# Bibliographical Notices

**Published:** 1839-07-06

**Authors:** 


### BIBLIOGRAPHICAL NOTICES.

*Dr. Hildreth's Address to the Medical Convention of Ohio, in the City of Cleveland, May 14th*, 1839.

In our last number we noticed the proceedings of the Medical Convention of Ohio. The address of Dr. Hildreth, the President of the Convention, upon the climate, and early history of diseases in Ohio soon after its settlement by the whites, is so interesting that we shall lay before our readers copious extracts from it. We much regret that a series of these histories of the early diseases of various parts of our country are not published,—they would enable us to judge correctly of the changes brought about by the cultivation and draining of land, and the superior degree of comfort and luxury which are obtained after the labours of clearing the forests and providing for the first necessaries of life.

Dr. Hildreth speaks of that part of Ohio which is contained within the limits of the Ohio Company's purchase, and is situated near the Ohio river. He divides his discourse under the following heads:

1st. Topography, and primitive aspect of the country on the Ohio river.

2d. Climate, and its changes from the effects of cultivation.

3d. Diseases of the aborigines.

4th. Diseases of the first white settlers, and early epidemics.

5th. Treatment of diseases thirty years since.

6th. Recent epidemics.

7th. Diseases common to this climate, with the modifications which have taken place from changes in diet, fashions, habits, &c.

8th. Closing remarks on the pleasures and privations of physicians.

The fourth, fifth, sixth, and seventh sections contain so much interesting matter, that we publish them entire. The remaining sections present less original matter, but are written in the same pleasing, unaffected style, and give us a most favourable idea of the excellence of heart, and of the clear, practical mind of Dr. Hildreth.

The country bordering upon the Ohio river was, like the rest of the United States, covered with an unbroken forest. The country is hilly, but is free from mountains and marshes; it was always remarkable for its great fertility, and previously to the settlement of the whites abounded with immense herds of deer and buffaloes.

The climate has, in some degree, been modified by the clearing away of the forests. The extremes of temperature and its sudden changes are greater, but the mean annual temperature is rather higher; the average of the last twenty years has been 50° of Fahrenheit. Very cold or very hot weather continues for a few days only; but the mercury has been known to rise in one instance as high as 99° in the shade, and to sink in another as low as 20° below zero. The mean annual height of the barometer is twenty-nine inches and fifty hundredths. It rarely runs to thirty inches, and in great changes only sinks to twenty-eight inches and sixty-nine hundredths. The variations of the barometer are extremely slight during the summer months. The coldest winds are those from the north and northwest; and when these winds are frequent in the summer after showers, the seasons are the most healthy. The reverse also holds good. December, May, and June, are the most healthy months; February, March, August, and September, the most sickly. The same remarks are true of the climate east of the mountains.

The information possessed by Dr. Hildreth relative to the diseases of the aborigines, seems to have been very limited, and he has judiciously confined himself to the few facts he could gather. Their treatment for small-pox consisted, it seems, in drinking an infusion of spruce, or of pine, while the patient was in a vapour bath, and then plunging him in cold water. This disease was excessively fatal amongst them. Hickory ashes, mixed in a little honey, was their remedy for worms. The Indians were, like all savage nations, subject to destructive epidemics, which desolated whole tribes, and almost depopulated large tracts of country.

"4th. *Diseases of the early Settlers of Ohio.*

When the Ohio Company first took possession of the country along the borders of the Ohio and Muskingum rivers, the whole face of the earth was covered with a thick growth of forest trees, which defended it from the sultry heats of summer, and moderated the rigors of winter. Yet, from the history of our climate as detailed in the former part of this discourse, we may suppose they suffered occasionally from the diseases common to both the tropical and the arctic regions. They sometimes were attacked with malignant remittents in the summer, and pneumonias and pleurisies in the winters, but no serious epidemics appeared until partial openings had been made in the primeval forests, and the wet, low grounds exposed to the action of a summer sun. Half-way business in cultivating and civilizing a country, is like half-way work in every other affair, often productive of evil. Accordingly, we find that a partially cultivated region is more sickly than one which is either totally covered with forests, or in a state of complete redemption. While it is shaded with trees, the swamps, and wet, low grounds, which more or less abound in all new countries, especially on the water courses, remain during the summer filled with water, and quite harmless; but as soon as the trees are cut away, and their trunks and branches left to moulder and decay in the heats of summer, the "*Hydra*" of the ancients is let loose, and all the arrows of *Apollo* can hardly slay the monster. The partially evaporated waters and reeking shores of the swamps, exhale a sickly malaria, which the morning fogs and the noon-day breezes waft to the cabin of the new settler, and his humble, but cheerful hearth, is soon clouded with sorrow, sickness, and pain. The hunters and wild borderers who preceded the actual settler, generally suffered much less from sickness. Their cabins were placed immediately on the banks of some river or creek. The clearing around their huts was generally very small—seldom more than an acre—a large portion of which would be shaded by the tall trees. Like the savages around them, the largest share of their time was spent in the forest, and the support of their families depended altogether on fishing and the fruits of the chase. Their diseases were few and simple. Since the first settlement of the country, many of the disorders have changed their type and character. From the year 1788, the period of the first improvements in Ohio, to the year 1807, the date of the first great epidemic, a large proportion of the diseases originated in exposure to wet, cold, hunger, and fatigue, and were generally of an inflammatory type, such as rheumatisms, pleurisies, pneumonias, scarlatina, and small-pox. Ophthalmias were also common. For the first nine years, the inhabitants made but little progress in clearing their lands of the huge forest trees which covered the rich alluvions on the Ohio and Muskingum rivers. The greater portion of their time and strength was occupied in building stockaded garrisons and blockhouses, and watching the movements of the Indians. Sometimes their lives were in danger from famine, and at others from the rifle and tomahawk of the savage.

In the spring and summer of the year 1790, the inhabitants of Washington county suffered severely from want of wholesome food. Very little land had as yet been cleared, and a severe and untimely frost in September of the preceding year, having destroyed or greatly damaged the crops of corn on the Monongahela, where they chiefly looked for their bread stuff, the settlements were on the brink of being ruined and broken up. The Indian war began the following year, and they still continued to suffer from want of food. The savages killed and drove away many of their cattle; and, continually watching in the vicinity of their garrisons, prevented the hunters from obtaining a supply of venison, which at that day were more numerous than the domestic cattle at this. In this season of want, I have heard some of our present inhabitants, who were then children, relate with what anxiety they watched from day to day the tardy growth of the corn, beans, and squashes, and with what rapture they partook of the first meal prepared from vegetables of their own raising.

In this period of time, namely, from 1790 to 1795, while confined in their garrisons, the settlements at Belleprie suffered much from small-pox and scarlatina. Of the latter disease many children died. Some families lost three or four. It was of a malignant character, and very fatal. The small-pox was rendered in a manner harmless by inoculation. Fevers of the remitting type were rarely seen, so long as the country was wholly covered with forests.

To counteract the depressing effects of want and anxiety on the mind as well as the body, all kinds of athletic amusements were encouraged by the colonists amongst the young people, especially foot races, games at ball, and dancing.

Some of the young females had become so habituated to danger, that nothing pleased them better than a sudden alarm that the Indians were about to attack them, as the confusion and bustle of such a crisis gave a different train to their thoughts, and a relief to the sameness of a garrison life. This volatility of spirits I have no doubt preserved the early inhabitants from many attacks of disease and death. The leaders of the colonists were generally officers and soldiers who had served during the revolutionary war, familiar with danger and the structure of the human mind.

Peace being again restored to the frontiers in 1795, by the victory of Gen. Wayne over the Indians, the colonists began soon after to sally forth from their garrisons, and, scattering over the face of the country, took possession of the wild or partially cleared lands which had fallen to their lots, before the breaking out of the war. In a few years extensive clearings were made, and large tracts laid open to the influence of the sun. Mill-dams were built, and abundant sources for origin of intermitting and remitting fevers created in the half-cleared lands, undrained swamps, decaying timber and weeds of the most luxuriant growth. All these, combined with the heats of summer, began to produce disease, and as autumn approached many pale faces were seen amongst these hardy adventurers. The disease was, however, seldom fatal; and a few simple remedies, with a more plentiful and nourishing diet, aided by the invigorating breezes of winter, soon restored their strength.

*Malignant fever at Galliopolis in the year* 1796. —This town was settled by a company of emigrants from France, in the year 1790. They had bought and paid for lands in their own country, from Joel Barlow, the agent of the "*Scioto Land Company*," which, failing to close its contract with the Congress of the United States for a large tract of wilderness lying between the Scioto river and that of the Ohio Company's purchase, could not fulfil the agreement with these men; and they were left in a strange land, without a home, and without the means of purchasing one, as their journey and payments to the Scioto Company had exhausted their money. With want, disappointment, and the Indians to contend with for several years, sickness and death would naturally fall upon them. During the Indian war, which broke out on their arrival, they were confined to their garrison, and could do but little towards clearing the lands on which they had been permitted to settle. Within the bounds of their village were numerous small ponds of water and wet low grounds, partially cleared and covered with weeds and decaying wood from the fallen trees. In the summer of 1796 a bilious remitting fever broke out which prostrated nearly the entire population and caused a number of deaths. Amongst them was a cousin of the writer of this address, who had early visited the west, and was engaged in the fur trade.

Andrew Ellicot, a celebrated engineer and surveyor, in a voyage down the Ohio river, landed there in November of that year. The following extract from his journal, is copied from the fourth volume of the Medical Repository, and is the only account of this sickness which I have been able to procure:

"Arrived at Galliopolis about eleven o'clock in the morning. The village is a few miles below the mouth of the Great Kenhaway, on the west side of the Ohio river, and situated on a high bank. It is inhabited by a number of miserable French families. Many of the inhabitants this season fell victims to the *yellow fever*. The mortal cases were generally attended with *black vomiting*. This disorder certainly originated in the town, and in all probability from an unusual quantity of animal and vegetable putrefaction in a number of small ponds and marshes within the village."

As this visit took place at a period when the yellow fever prevailed as an epidemic in many of our commercial cities, and great disputes were carried on amongst the medical men, as to its contagious or non-contagious nature, he goes on further to state: "The fever could not have been taken there from the Atlantic states, as my boat was the first that descended the river after the fall of the water in the spring; neither could it have been taken from New Orleans, as there is no communication at that season of the year, up the river. Moreover, the distance is so great that a boat would not have time to ascend the river after the disorder appeared that year in New Orleans, before the winter would set in." From this we learn that the fever was of local origin, and also that it took four months to perform a voyage from New Orleans, which is now accomplished in ten or twelve days.

The next serious sickness, in point of time, took place in the Scioto valley.

*Epidemic fever at Chillicothe, and the valley of Scioto river*, in the years 1800 and 1801; described by Dr. Harrison in the 10th volume of the Medical Repository.

The town of Chillicothe was first settled in the year 1796, under the auspices of Gen. Massie; and remained healthy, with the exception of slight intermittents to the year 1800. The summer of this year, until about the middle of August was dry, and the inhabitants tolerably healthy. Near the middle of this month, a heavy rain filled the streams so as to overflow their banks. The water was of the color of the ley of wood ashes, and emitted a nauseating smell. The country was new, and the cultivated lands but partially cleared. In a few days after this rise of the river, a large proportion of the inhabitants of the town fell sick with bilious fever, generally of the remitting type, and of great severity. About the same time a similar fever appeared in all the principal settlements in the valley of the Scioto. Several, he says, turned yellow after death, and exhibited early marks of putrescency. The fever abated in October. In the following winter there was very little snow, but much rain, and the spring of 1801 was unusually wet; the streams overflowed their banks and inundated the bottoms as late as the month of June. From this period to the last of August there was a great deficiency of rain, and a severe drought followed, attended with winds from the north-west, north, and east. Towards the close of this month there were abundant showers. September was as hot as any of the summer months. The wheat crop in many places was attacked with "*rust*," or vegetable mould, producing a diseased kernel, well known to the early settlers by the very appropriate name of "sick wheat;" and causing nausea and vomiting in those who ate of the bread made of this kind of grain. It is no doubt occasioned by the same malarious condition of the air, which produces sickness in man, as it is known to prevail most in such summers. This year the fever commenced early in July on Deer Creek, being one of the most fatal localities in the preceding season; and soon after at the other settlements, especially the "Pickaway Plains," an extensive and very rich prairie, a little south of the present town of Circleville, and as noted in its vicinity, in these early days, for intermittent and remittent fevers, as the "Pontine marshes." In Chillicothe the disease commenced the beginning of August, and Dr. Harrison was one of the first attacked, as if the "Hydra" was fearful of his interference in the operations of this season. Cases of the fever were not common in the town until the middle of the month; from which time it prevailed generally until the last of October. This year, however, it was less fatal in town than in the country.

The symptoms which attended this epidemic, were similar to those which usually attend bilious remittents, and are minutely described by Dr. Harrison. It attacked all ages and sexes, but was more fatal to females than to males. Colored persons were not more exempt from attacks than the whites. The effects of the poisonous malaria, says Dr. H. were not confined to man, but also showed its sickly influence on domestic animals. Horned cattle suffered from diarrhœa and bloody murrain. Horses, from "yellow water," a species of jaundice; and more than all from an ulcerated and aphthous state of the mouth, which in many instances proved fatal.

In the year 1815, many horses suffered from a similar complaint in Washington County, and especially amongst the horses attached to the army in the vicinity of Upper Sandusky this disease was very severe. I have also heard of geese and dogs, in these sickly seasons, having regular paroxysms of chill and fever—which I have myself seen.

*History of the diseases of the early settlers, concluded.*—At the first settlement of the country, consumption, or Phthisis pulmonalis, was a disease nearly unknown to the inhabitants. The invigorating effects of constant exercise in the open air, exposure to all kinds of weather, a simple but nourishing diet, and the enlivening faculties of the mind kept constantly in play, forbade the approach of this scourge of indolence and the refinement of modern life. Very few cases occurred until after the epidemic of the year 1807, and these did not then average more than one death a year in a population of two thousand. Since the year 1815 and 1816, when Pneumonia Typhoides prevailed, consumption has been gradually increasing, and at this time the average annual amount is about three deaths in a thousand inhabitants, having increased in the course of forty years in a very rapid ratio.

*Epidemic fever of* 1807.—It is now more than thirty-two years since I commenced the practice of medicine in Ohio, having emigrated from the state of Massachusetts in the year 1806, at the age of twenty-three years. Early in October I reached Marietta, and remained about two months. In December, settled at Belleprie, twelve miles below, and there exercised my profession through the epidemic of 1807, and returned to Marietta in March, 1808, where I have since resided.

*Natural Phenomena which Preceded and Attended the Epidemic.*—Some part of the winter which preceded the great epidemic of 1807, was remarkable for the intensity of the cold. In February, after the fall of a few inches of snow, the Ohio river was frozen across so firmly in one night, that loaded wagons crossed the next day on the ice. The summer of 1836 was dry and warm, and rendered extraordinary by the ravages of the "army worm," as it was very appropriately called by the inhabitants. It is a worm about two inches long, dark coloured, smooth cuticle, with two light coloured stripes running the length of the back, and is the larva of an ash coloured moth, the specific name of which I do not know. Their numbers were without limit, covering the face of the earth, and moving along like an army, when changing their quarters in search of nourishment. The cereal grapes and grains were their favourite food, but their chief supplies were obtained from the leaves of the forest trees, as there were then but comparatively few cultivated spots. Some persons preserved their fields by digging ditches in the dry earth, into which they tumbled and perished by millions. Like the frogs of Egypt, they invaded and traversed through dwelling houses, and in one cabin, a few miles above Marietta, actually drove out the occupants, and obliged them to take shelter under a large tree for several days. They made their appearance the last of April, and disappeared the first of June. Since then the same worm has been seen in limited districts, but not in such myriads, nor over such a wide extent of country. The spring of 1806 was very early; many peach trees were in blossom by the 25th of February, and all of them by the middle of March. That of 1807 was more backward than usual, and uncommonly wet. The summer was not less so, and every fair day was preceded and followed by two or three wet ones. The heat was not greater than common, the mercury seldom rising above ninety degrees. Books and furniture were covered with mould, and every farmer lost more or less of his grain, hay, &c., from lack of sunshine to dry them. There was not less than three freshets in the Ohio river. The low lands were covered with water, and much corn and grass destroyed.

*Approach of the Disease.*—The inhabitants, from their location on the bottoms, were generally living in the vicinity of stagnant water, and of course the larger number of them were attacked with the disease. The months of February and March were attended with catarrhal fevers of great obstinacy and severity. It was noticed that no one who had been affected with this disorder was attacked with the fever of the same summer and autumn. In April, May, and June, no particular sickness prevailed, but there were many cases of ophthalmia. A school of small children was broken up for a few days, in consequence of its attacks on the scholars. This disease was much more common thirty years ago than it is now, and probably arose from a peculiar condition of the atmosphere. By the middle of July, intermittent and remittent fevers were common. In August, scarcely a family in the township was free of the disease in some form or other. It extended both up and down the Ohio river for several hundred miles, but was chiefly confined to the alluvions and low lands; the inhabitants of the more elevated and hilly parts of the country suffering but little from the fever.

It began earlier at Galliopolis and several places below Belleprie, and was much more mortal. At Marietta the epidemic was very fatal; more than fifty dying in the course of the summer—while at Belleprie, out of nearly two hundred cases, there were but four or five deaths.

The disease appeared in various grades of intensity, from that of a mild intermittent, to the worst form of bilious remittent—in some cases resembling very nearly the yellow fever of the Atlantic cities. The duration of the remitting form was from five to seventeen days, unless interrupted by medicine. It ceased with the first heavy frosts.

The Influenza visited all the western country in the autumn of the same year. It began in August, in the Atlantic states, but did not reach the Ohio till October, and passed away early in November.

At the setting in of cold weather several cases of pneumonia typhoides occured, very similar in type to the epidemic disease of 1815 and 16. These cases were treated successfully by stimulants and blisters.

*Diseases from* 1807 *to* 1822.—The winter following the epidemic of 1807, was mild, and the summer months were marked with no prevailing disease. From the year 1807 to 1813, the country was very healthy. The few fevers which did appear were generally typhoid, or synochal. Bilious cholics, for several years after the epidemic, was a very common disorder. Phthisis pulmonalis also became rather more frequent after the influenza, but was still a rare occurrence. During the heats of summer, cholera infantum was greatly more frequent than it is at present, and often proved fatal. It probably arose from the same malarious state of the atmosphere which produces intermitting fevers, as we find it most prevalent in regions favourable to the latter disease.

In 1810 and 1811, an epidemic rabies appeared amongst the dogs, wolves, and foxes. Many domestic animals were bitten and died of hydrophobia. Several persons were bitten, but I do not recollect any death from this cause. One case came under my care, attended with all the usual symptoms of the disease, and was promptly arrested by the free internal use of calomel and cantharides; producing strangury and ptyalism in a few hours.

In the years 1813, 14, and 15, typhus fevers were common in the summer and autumn; and but few cases of well marked bilious intermitting or remitting fever appearing in all the period from 1807 to 1817. Pneumonia typhoides, or "cold-plague," as it was vulgarly called, was prevalent in the eastern states in the years 1812, 13, and 14; but did not reach this country until the winter and spring of 1815 and 16. Uncommonly cold winters had preceded the disease, the debilitating effects of which were supposed to have been the predisposing cause. The winters with us being milder, might have had some effect in ameliorating the attacks, as they were generally less fatal than at the east; but this, however, is mere conjecture, as the real source of all wide spreading epidemics is yet clouded in mystery.

In the winter of 1816, it proved very mortal in some parts of the country. With us, it prevailed most in the highlands, and more rarely in the bottoms. The dread of this disease amongst the people was very similar to that felt a few years since on the introduction of the cholera, and attended with the same diversity of opinion as to its contagious or non-contagious character. Even the name, "*cold-plague*," struck a chill to the heart. Aided by the experience of the eastern physicians, and the pathology of the epidemic being better known, a large proportion of the cases were under the control of medicine. It was most fatal amongst our soldiers on the frontiers, during the late war, and in many localities truly merited the name of "plague." From the year 1817 to 1820 no general disease prevailed. The fevers of summer were commonly synochal, or of a sub-typhoid type, and during winter inflammatory.

*Epidemics of* 1822 *and* 1823.—We now approach the period of the great epidemic fever which prevailed in the valley of the Ohio, from the year 1821 to 1824; but was most rife with us in the years 1822 and 1823. The summer of 1821 was very sickly in some portions of the country, especially at Louisville, Ky., so that there seemed to be a change taking place in the constitution of the atmosphere, preparatory to the great epidemic which followed.

These fatal seasons will long be remembered in the annals of this portion of Ohio, as the most disastrous of any since its first settlement.

*Natural Phenomena which Preceded or Attended the Epidemic.*—The years 1820 and 1821 were distinguished by unusual drought; the summers hot, and little or no thunder or brisk gales of wind to purify the atmosphere. The rivers and creeks were low, and in the summer and autumn of 1822 the Ohio was lower than ever before seen by the oldest inhabitants. The water in the latter stream was nearly stagnant, excepting at the heads of the islands, resembling a long lake more than a river, and bearing on its surface numerous patches of a mucous scum or froth. The shores and shallows were lined with aquatic plants and grasses for many rods in width, as early as the fore part of June, whereas in common seasons they do not appear sooner than August or September, and coated with a green vegetable matter, *Lemna minor*, which, as the water gradually subsided, or the winds forced it on the shore, remained on the beach exposed to the sun. These manufactories of poisonous gases, together with the stagnant waters, exhaled a noisome smell, which was noticed by the inhabitants, especially in the morning and evening, and at all times of the day by persons living on the uplands, beyond the immediate influence of the miasm, who came to the banks of the river on business, or to visit their sick friends; the course of the wind, for four months, was, with little variation, from the south and south-west—all which, combined with the previous epidemic constitution of the air, must have aided in producing the sickness. The unremitting warmth and aridity of the weather, proved wonderfully favourable to the production of insect life, especially so to the caterpillar families. Every wild cherry tree, black, and white walnut, with various other trees, were so much stripped of their foliage, and so covered with the webs of the insects, as to change their usual verdant appearance to that of grey or white.

Many other genera of insects also abounded. The grass-hopper, *Gryllus telligonia*, in many districts were so numerous as to destroy whole fields of oats and grass in the course of two or three days; rising in clouds when disturbed, and making a rustling with their wings, which afforded a striking resemblance to the locust of the eastern continent.

The potato-fly, *Cantharis vittata*, was generated in such multitudes, that a pint cup full could be gathered from a single hill of potatoes; and many pounds were collected, and used with as much vesicating effect as the best officinal cantharides. In common seasons they are rather a rare insect in this region of country, and have not appeared in any quantity since that time.

Animalcula, peculiar to the human family, were generated in a wonderful manner; and an acquaintance of mine, of cleanly habits, who wore a flannel vest next his skin for a few days longer than usual without changing it, was much confounded and chagrined to find it thickly inhabited with lice.

In September the country was overrun with myriads of gray squirrels; they travelled in armies, and destroyed many fields of Indian corn, especially in the newer clearings, bordered with trees. Their course of travel was from the north to the south. No obstacle checked their progress; passing through or over dwelling houses, and crossing the Ohio river by swimming. Its low stage was favourable for this purpose. It is remarkable that the same migrating propensity was upon them in the epidemic autumn of the year 1807. It has been noticed as early as the days of Hippocrates, that great and extensive epidemics are often attended with other unusual and remarkable phenomena, which either precede as harbingers, or accompany the progress of the disease. Many curious facts of this kind have been collected by the learned Noah Webster, in his laborious and unique work on "Pestilence."

*Commencement and Progress of the Epidemic in* 1822.—The first cases appeared in the latter part of June. They were generally typhoid, and attended with signs of malignity, such as petechia and glandular swellings, most generally of the parotids. If suppuration took place, the patient generally recovered; if otherwise, the disease was usually fatal.

The fore part of July was damp and cool, soon after which the fever put on the remitting form, and no more petechial cases occurred. By the first of August, the epidemic was general in this portion of the valley, and especially in Marietta. The largest number of attacks was in September, and at one time there was not less than four hundred cases within the area of one square mile. They were composed of all, from the mild intermittent to the most malignant remittent, with the usual symptoms which attend the yellow fever, During the season I had about six hundred patients under my care. For four months in succession, I ate but two meals a day, and spent from sixteen to eighteen hours out of every twenty-four in attending on the sick. Through a merciful Providence, my own health was good, and the only suffering was from exhaustion and fatigue, through the whole of this disastrous season. The proportion of deaths was about six in every hundred cases, where proper medical attention was given to the sick; but so general was the disease, that many lives were lost from a lack of nurses. All other disorders were swallowed up by this. It did not sensibly abate until after smart frosts appeared in November. Intermittents were common through the winter months.

*Symptoms Peculiar to the Epidemic of* 1822.—The general features of the disease were such as are common to bilious remittents in other parts of the world; but it possessed others which were peculiar to that of this year. Amongst these was involuntary and deep sighing. It was so general, that you rarely approached a patient who was very sick, and asked any question, who did not, before he answered, draw one or two profound sighs. This symptom denoted congestion, and is usually a mark of danger, although they generally recovered in whom it appeared. Yellowness of the eyes and skin, first appearing on the forehead and neck; from whence it gradually spread over the whole body. A cold, clammy state of the surface, and especially of the lower extremities, when at the same time the patient complained of uncomfortable heat, and begged to be bathed in cold water. In many of the fatal cases, coma attended for thirty or forty hours before death. All ages were attacked, but it was seldom fatal to children under four years old. More deaths took place on the fourth, seventh, and ninth days, than at later periods, as the disease had then spent its force, and was under the control of medicine.

*Character and progress of the epidemic in the year* 1823.—The fore part of the winter of 1823 was very wet, but the weather was mild, and the inhabitants generally healthy. The spring months were pleasant, with no unusual indications of a sickly season. But in June the fever again broke out and pervaded nearly all parts of the country in the course of the summer. It was not now, as in the past year, chiefly confined to the vicinity of the water courses and rich alluvions, but infested the uplands equally with the valleys. Even some districts within the ranges of the Alleghany mountains were visited with intermitting and remitting fevers. East of the Alleghanies, in Pennsylvania, it was very prevalent in many places not only in 1823, but also in 1824.

The country along the valley of the Ohio river was deluged with rain, in June and July, attended with very little thunder and no strong winds. It rained on fifteen days in the month of July, and in the course of these two months there fell fifteen inches of water. All the low grounds and hollows were filled with it, and exhaled noxious and fœtid vapours; in many places so disgusting to the smell, that persons in passing over or near them, involuntarily put their hands to their noses, and made all haste beyond their influence. With a light moderate breeze passing from the low lands to the hills, the same sickly vapour could be smelt a long distance from its source. Even the flat lands on the hills, if covered with a coat of grass, exhaled the same nauseous gases. It was noticed by observing farmers, that in plowing the corn lands, the earth, instead of the pleasant odour which arises from fresh plowed soils, sent forth a disgusting smell. The surface of the ground seemed to be in a putrid state, from the lack of sunshine to dry and sweeten it. Many fields of Indian corn were damaged by the excessive wet state of the low grounds, and much wheat was lost after passing through the hands of the reaper. Grass suffered in the same way. The last of August the rains ceased, and the weather was more dry the residue of the season; but this change had no influence in checking the progress of the epidemic.

In the months of September and October, dysenteries were common, and often alternated with intermittent fever in the same subject. In other portions of the country, on the hills, dysentery appeared as an idiopathic disease.

The fever of this year was of a similar character with that of 1822, but bore evacuants better, so much so that bleeding was decidedly advantageous in a great many cases. In proportion to the number attacked, the disease was more fatal in the country than in the town, especially among amongst those living in the wide rich bottom lands on the Ohio river. This was doubtless occasioned by the stagnant water around them, and the luxuriant growth of weeds which clothed their farms from the lack of able bodied persons to till them—thereby obstructing the circulation of the air, and by their decay filling it with noxious gases. Never did the eye behold such an enormous growth of weeds as had taken possession of the rich lands, rising in some fields to the height of fifteen feet. "The earth brought forth by handfuls," in every spot not drowned by the rains. The orchards were loaded with fruit, and the woodlands with all kinds of nuts and acorns. The fields were burthened with grain and grass, and unchecked by the weeds, the Indian corn raised aloft its spiry head and produced an abundant crop—in many instances without any further aid from man than only once plowing and planting. Had not an overruling and kind Providence interposed, famine must have followed the sickness, as is common in other countries, for there were not well people enough to take care of the sick, much less to attend to the cultivation of their farms.

Some estimate may be formed of the condition of the country, when it is stated that at the election for state officers, the county of Washington gave but three hundred and ninety votes, in place of the twelve or fifteen hundred usually polled.

Instead of the storms of rain incident to the equinox, the wind was from the north, the weather fair and cool, and the nights of the twentieth, twenty-first and twenty-second of September, accompanied by a smart white frost, sufficient to produce ice of the thickness of window glass. So far from checking the epidemic, the attacks were more freqent after this cool temperature, and not less malignant. The disease was not subdued until the setting in of hard frosts, the beginning of November. After this period no new cases of remittent appeared; but intermittents and some cases of dysentery continued to harass the people for some weeks, as if the "malarious hydra" was loath to quit the terrritory over which he had so long reigned.

5*th. Treatment of diseases in Ohio* 30 *years ago, and especially the epidemic of* 1822 *and* 1823.

The epidemic fever of the year 1807 was of a more inflammatory type than that of 1822 and 1823, and bore free evacuations better, especially that of bleeding. Our doses of calomel at that period were small; five grains was a medium dose, combined with twenty grains of jalap for a cathartic. This quantity generally produced the desired effect, and was found to be one of the most useful purges. Our febrifuge powders were composed of nitrate of potash, with some preparation of antimony, generally the tartrate. The "neutral mixture" was also considerably used; but a favourite medicine, especially in fevers of a malignant type, was a solution of the sub-carbonate of soda. It had come into favour under the auspices of Dr. Samuel Mitchell, of New York, the senior editor of the Medical Repository, a work of much merit for that day. His theory was, that malignant fevers were caused or greatly aggravated by the generation of septic, or nitrous acid in the stomach and bowels, which by a free use of alkalies was neutralised, and the fever rendered mild and more manageable. Whether the septic acid or acid of the putrefaction is more or less abundant in fevers, I do not know, but of this I am certain, that in the fever of 1807 alkalies proved to be one of our most useful and salutary remedies, and was also equally beneficial in the epidemic of 1822 and 1823. It was used not only internally, but in bad cases externally, combined with spirit and water, to which we sometimes added the capsules of the red pepper. By keeping the patient wrapped in a sheet thoroughly soaked with this solution, I have seen some of the most malignant cases of fever arrested and cured with little or no internal means, except a cathartic. It seemed to neutralise and destroy the fomites and seeds of the disease. As a tonic, Peruvian bark, in substance, mingled with a decoction of *Radix serpentaria virg.* was our chief reliance—sulphate of quinine and its numerous relatives were not known in Ohio until the period of the close of the second epidemic in 1823. In the malignant cases of that period, in addition to other remedies, was added that of yeast and charcoal in fine powder. It was a most admirable medicine where it could be swallowed in sufficient quantities and was nicely prepared. With this simple compound, which every cabin could furnish, I have seen the worst cases of fever subdued in thirty-six or eight and forty hours; our main hope, however, was placed on the curative action of mercury, in its various forms, especially the sub-muriate. If the specific effects of this wonderful medicine could be brought to act on the liver and secretory tissues, the patient was safe; and this could in most of the cases be effected, if he could bear bleeding. In some, however, no such action could be produced, and the disease was then generally fatal. For subduing intermittents, "Fowler's solution" was equal to any other remedy.

6*th*. *Epidemics from the year* 1824 *to* 1838.

From the year 1824 to 1826 no general disease prevailed. A few cases of measles and scarlatina appeared in 1824 and 1825. In February, 1826, the influenza passed over this part of Ohio, on its way westward. It began east of the mountains some weeks earlier. The attacks were less general in the country than in cities. In New York it was estimated that there were not less than twenty thousand persons sick with this disease at one time. I knew of no fatal case with us, though some of them were very severe, as was experiened in my own person. It differed in some of the symptoms from that of 1807; there was less affection of the lungs, and more of the frontal sinuses and head. From this period to 1832, no general disease prevailed. Early in that year the people began to be alarmed with the accounts from Europe of the ravages of the Asiatic Cholera, which occupied all the newspapers; and it made its appearance on the N. E. coast of America about the last of May, and spread with fatal rapidity along the great water courses which border the northern side of the United States. At Quebec and Montreal its mortality was frightful in June and July. It seemed this year to follow the course of the great lakes, in about the same parallel in which it crossed the Atlantic, and by September had reached some of our military outposts on the upper Mississippi. A lateral branch from the malarious column seems to have been thrown off southerly, and passed down the waters of the Hudson river to Albany and New York, where it appeared early in July. With us no cases occurred this year, but a few appeared late in the season at Cincinnati, and two or three persons were buried on an island in the Ohio river near Marietta, who had been attacked with the disease and died on their passage up, on board a steamboat. Either from a nervous dread of the disease, or some morbific constitution of the atmosphere, a large majority of the inhabitants this summer were troubled with bowel complaints, generally a moderate diarrhœa. In some it was attended with severe pain and constipation like a cholic. No disease which ever visited the civilized world ever held such control over the nervous system and moral faculties of man; and during the period when the great mass of our citizens believed it to be contagious, I have no doubt that one half of its victims took the disease, and actually died from the depressing effects of despair and fear. I know one individual, in whom, from the first, the greatest horror was awakened at the name of the disease, and who was attacked with a diarrhœa while it was raging in Montreal. It continued to follow him through the two succeeding seasons, and he has only been free from the disease since the cholera has left the country. Had it prevailed with us as it did in many other places, he would have been one of its earliest victims, but happily it did not. The same man was sick in 1822 and 23 with the prevailing epidemic, and was cured as much by the stimulus of hope, and the confident assurances of his recovery by the physician, as by the medicine administered. The same salutary effect was often produced by the specifics and nostrums vended during the cholera. The full confidence reposed in the remedy, from the printed certificates which accompanied it, often doing more in curing the patient than the medicine itself. In 1833 and 1834 this epidemic scourge still continued to visit our most populous towns and cities in the west, while the sparse and thinly settled portions of the country scarcely felt its effects; as if the appetite of the demon could only be satisfied with a multitude of victims, crowded into a small compass, and a short space of time. Those places and localities the most favourable to malarious diseases, seem generally to have suffered the most severely from its visits, showing that the same laws which govern other epidemics, also governed this, and as, *à priori*, we should be led to anticipate, I think it was found that the cleanly and well ventilated portions of our cities suffered the least, and the filthy and ill-aired the most. Since the year 1835 but few cases of cholera have been seen, although it is probable that in the hot and wet portions of the summer, sporadic cases may for some years occasionally appear. During its continuance in the country, even in districts where no cases of cholera appeared, it was observed in treating our summer fevers, that the greatest caution was needed in the use of cathartics, as even moderate doses often produced very drastic effects. This peculiar irritability of the bowels, showed the vast and overpowering influence of the "choleric malaria," in its controlling effects on other diseases, as is always the fact in great epidemics. Another evidence of its being governed by the same laws as malarious diseases, is the fact of its ceasing to prevail in any district, soon after the setting in of hard frosts. This was the fact in America, although it is said to have been very fatal in Russia during some of the winter months, soon after its first introduction into that country.

*7th. Diseases common to this climate, with the modifications which have taken place from the effects of epidemics, diet, fashions, &c.*

Immediately after, and for a few years subsequent to the epidemic of 1822 and 23, enlarged spleens, diseased livers and dyspepsia were far more common complaints than previous to that time, and often in persons who had escaped an attack of the fever; thereby indicating that the seeds of the epidemic had pervaded the system of every one, showing it in the subsequent effects. Dropsies have also been of more freqent occurrence. Measles and scarlatina have generally prevailed at intervals of eight and ten years, but have been more rife in 1838 than at any other period. Whooping cough is a more frequent visiter, and may be expected every four or five years. While speaking of this disease, which is often the "opprobrium medicinæ," I cannot forbear noticing a very old fashioned remedy which has been far more successful in my hands than any or all the new ones. It may be because I am partial to old fashions. This medicine is the sulphuret of potash, given in honey, three times a day, in five or twenty grain doses, according to the age, after suitable evacuants. It seldom fails to cure in ten or twelve days. Bilious cholics are more rare than formerly. Calculous affections are not common in this portion of the state. Apoplexies and palsies are of infrequent occurrence, and more especially so, since the use of alcoholic drinks have gone out of fashion as a common beverage. In the winter and spring months, cynanche trachealis is a frequent disease amongst children. Rheumatism is not very common; but Pneumonia and Pleuritis are the most frequent diseases in the winter and spring months, which doubtless arises from the great and sudden changes of temperature peculiar to this climate. We can hope for no alleviation in these diseases, but from a greater attention to our own persons, by accommodating our dress to the weather. Verminous diseases and cholera infantum are less frequent than formerly, and may in part be attributed to a more suitable diet and comfortable clothing, but chiefly to a more healthy condition of the atmosphere. The early decay of the human teeth is a complaint, often heard, and is well worth the attention of the medical faculty, to learn the cause and point out a remedy. Puerperal fevers are less frequent in the country than in crowded cities. I have seen but a very few cases in the course of my medical life. The "milk sickness," so much dreaded by the early settlers on the waters of the Miami, and the country between that and the Scioto, has never been known as a disease in the hilly regions east of the latter river. In the year 1807, there was some indication of this disease along the Ohio river, at Belleprie and vicinity, and many people believed that the milk used at their meals, during the epidemic fever, made them sick. Some of the cheese made on a large dairy farm, occasioned vomiting in every one who ate of it.

In 1822 and 23, the same fault was found with the milk in several places; but it passed away with the prevailing epidemic, and has not been noticed since. From these simple facts I am led to adopt the theory that malaria is the cause of this deleterious quality in the flesh and milk of domestic animals, and not any poisonous plants. Nervous complaints and consumptions have increased many fold, within thirty years, and have chiefly arisen from the more luxurious and sedentary habits of the people, especially our females. The low price of factory cottons and cloths having banished the distaff, spinning-wheel and loom, from our houses, has, without doubt, largely contributed to this result. Formerly, the greater portion of our clothing was made within our own dwellings, and the music of the spinning-wheel, accompanied by the cheerful female voice, instead of the piano, could be heard in every house. The latter may please the ear for a moment, but no permanent result remains; while with the music of the wheel we have rosy health, vigorous and actixe frames, and garments to clothe the beloved ones of our own households, as well as the needy poor. It is sadly apparent that with the passing away of our domestic manufacturers, there also departed a large share of the health, and much of the happiness of our females.

We regret that we have not room for the closing remarks of Dr. Hildreth: though not immediately connected with medicine, they relate to what is, at least, as interesting—the self-sacrifices and arduous duties of a physician. For these the compensation he receives is most inadequate,—

in the words of Dr. Hildreth, "The pleasing thought that he has been the blessed means of rescuing a father, a mother, a child of some distressed family from the grasp of death, must ever remain his richest compensation, to be prized far above gold, however necessary it may be to our wants, and is the best, if not often his only reward, for many a long and weary ride, and many a sleepless night."

If the Medical Convention of Ohio gives rise to many papers as interesting as that of Dr. Hildreth, its labours must not only advance the state of science in Ohio, but it will ultimately eradicate the charlatanism which, under the guise of a system of treatment, has infected the Union, and had apparently taken deep root in that state. We have no doubt of the good that may be effected by well-organized conventions, bringing together the physicians from various parts of a state, and directing their united efforts to the study of the diseases which are most rife in the country in which they practise. No attempt of the kind has yet been made in Pennsylvania, or in many other states; there are, as yet, many difficulties in the way, but we doubt not that they might readily be overcome,—and the advantages which would result from such a meeting, would probably become the permanent success of these scientific meetings.

---

*A New Dictionary of Medical Science, Second Edition, with numerous Modifications and Additions.* By Robley Dunglison, M.D., M.A.P.S., &c. 8vo. pp. 821. Philadelphia: Lea and Blanchard. 1839.

So well known are the merits of this valuable work, that, in noticing a second edition of it, it will suffice to extract the remark of the author in the preface, that "it will be found to contain many hundred terms more than the first, and to have experienced numerous additions and modifications." It has been got up by the publishers in very handsome style, and must command, as it deserves, an extended circulation.

